# Hydroxide Conduction Enhancement of Chitosan Membranes by Functionalized MXene

**DOI:** 10.3390/ma11112335

**Published:** 2018-11-21

**Authors:** Lina Wang, Benbing Shi

**Affiliations:** 1 College of Chemistry and Chemical Engineering, Xi’an University of Science and Technology, Xi’an 710054, China; 2 School of Chemical Engineering & Technology, Tianjin University, Tianjin 300072, China

**Keywords:** functionalized MXene, chitosan, anion exchange membrane, hydroxide conductivity, alkaline fuel cell

## Abstract

In this study, imidazolium brushes tethered by –NH_2_-containing ligands were grafted onto the surface of a 2D material, MXene, using precipitation polymerization followed by quaternization. Functionalized MXene was embedded into chitosan matrix to prepare a hybrid alkaline anion exchange membrane. Due to high interfacial compatibility, functionalized MXene was homogeneously dispersed in chitosan matrix, generating continuous ion conduction channels and then greatly enhancing OH^−^ conduction property (up to 172%). The ability and mechanism of OH^−^ conduction in the membrane were elaborated based on systematic tests. The mechanical-thermal stability and swelling resistance of the membrane were evidently augmented. Therefore, it is a promising anion exchange membrane for alkaline fuel cell application.

## 1. Introduction

As an efficient, clean, and zero-emission power generation technology, fuel cells can effectively solve energy crises and environmental pollution issues as the focus of global governments. Compared with other types of fuel cells, the ones with alkaline anion exchange membranes have attracted wide attention because of: (1) Low working temperature (60–90 °C); (2) high rate of oxygen reduction reaction and catalytic stability, which can decrease the Pt amount or use non-noble metal catalysts such as Ag and Ni instead, thus significantly reducing the cost; (3) low fuel permeability (permeability of methanol: <10^−6^ m^−2^ s); and (4) facile water and heat management [1,2,3]. Alkaline anion exchange membranes are one of the essential components of alkaline fuel cells and are capable of blocking fuel and conducting OH^−^. Its performance, especially OH^−^ conductivity, directly determines the open circuit voltage and energy output of fuel cells. However, currently available alkaline membranes all have low OH^−^ conductivities owing to a low diffusion coefficient of OH^−^ [4] (movement rate of OH^−^ is 56% of that of H^+^) and poor channel connectivity inside. Thus, the commercialization of fuel cells has been seriously limited.

Alkaline anion exchange membranes are mainly prepared by functionalized polymers, including polyphenyl ether [5], polybenzimidazole [6], and polyetheretherketone [7]. OH^−^ is mainly conducted through conductible cation aggregation zones inside membranes, so the continuity of these zones plays a decisive role. Since traditional polymers have similar functionalized and non-functionalized areas, continuous ion conduction channels cannot form through self-assembly [8,9]. Recently, block polymers have been prepared to significantly increase the OH^−^ conductivity of membranes through self-assembly-generated interconnected ion cluster channels due to entropy-driven microphase separation of hydrophilic and hydrophobic chain segments. Nevertheless, the preparation processes for block polymers are complicated and the distribution of groups cannot be accurately controlled, so it is difficult and non-universal to generate interconnected conduction channels depending on functionalized polymers [10].

Comparatively, organic–inorganic hybridization is a convenient and effective strategy to elevate the ion conduction performance of membranes. On one hand, the surface of functionalized inorganic nano-filler allows formation of continuous ion conduction channels. On the other hand, adding functionalized nano-filler can connect non-conduction dead zones, thereby significantly raising the conductivity [11,12]. In addition, addition of inorganic nano-filler can markedly augment the thermal stability and mechanical performance of membranes. At present, inorganic nano-fillers are mainly structurally classified into: (1) Zero-dimensional nanoparticles (SiO_2_ [13], TiO_2_ [14], etc.), (2) one-dimensional nanotubes (halloysite nanotubes [15], carbon nanotubes [16], etc.), and (3) two-dimensional nanolamellar materials (graphene oxide [17], hydrotalcite [18], montmorillonite [19], MoS_2_ [20], etc.). Two-dimensional nanolamellar materials, which have high specific surface areas that benefit the construction of long-range and wide continuous channels, are thus superior to other materials [21]. Nevertheless, routine two-dimensional nanolamellar materials have limited functions owing to low contents and uneven distribution of surface functional groups. Thus, it is imperative to develop novel functionalized two-dimensional nanolamellar materials for preparing hybrid anion exchange membranes.

In this study, we selected a new lamellar material rich in oxygen-containing groups, MXene, to graft imidazolium brushes through precipitation polymerization followed by quaternization. Afterwards, we fabricated a hybrid membrane by using functionalized MXene and chitosan matrix with excellent film-forming ability. Moreover, we systematically studied the construction of interfacial conduction channels and the mechanism for ion conduction by characterizing the microstructure and testing related properties.


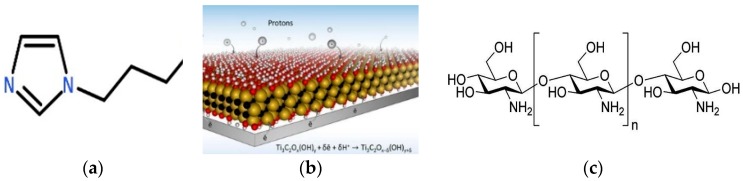


## 2. Experimental Section

### 2.1. Materials

Titanium hydride (TiH_2_, 99%, 325-mesh), titanium carbide (TiC, 99%, 325-mesh), aluminum powders (Al, 99%, 325 mesh), and 1-vinylimidazole were purchased from Alfa Aesar (Shanghai, China). Hydrofluoric acid (HF, 49%) was bought from Aladdin Reagent Inc. (Shanghai, China). Dimethyl sulfoxide (DMSO, analytic grade), glacial acetic acid (analytic grade), and potassium hydroxide (analytic grade) were obtained from Tianjin Kermel Chemical Reagent Co., Ltd. (Tianjin, China). CS (degree of deacetylation: 95%) was provided by Zhejiang Golden-Shell Pharmaceutical Co., Ltd. (Yuhuan, Zhejiang, China).

### 2.2. Apparatus

A TEM-100CX transmission electron microscope was purchased from JEOL (Boston, MA, USA). An FTIR Thermo Nicolet IR200 Fourier transform infrared spectroscope was bought from Thermo Scientific (Waltham, MA, USA). A D8 Advance ECO X-ray diffractometer was obtained from Bruker (Karlsruhe, Germany). A TGA-50 thermal gravimetric analyzer was provided by Shimadzu (Kyoto, Japan). An M350 AX all-purpose tensile testing machine was purchased from Testometric (London, UK). A CHI660B electrochemical workstation was bought from Zhengzhou Shiruisi Instrument Technology Co., Ltd. (Zhengzhou, China). A KJ-1600G muffle furnace was supplied by Zhengzhou Kejia Furnace Co., Ltd. (Zhengzhou, China).

### 2.3. Methods

**Figure 1:**
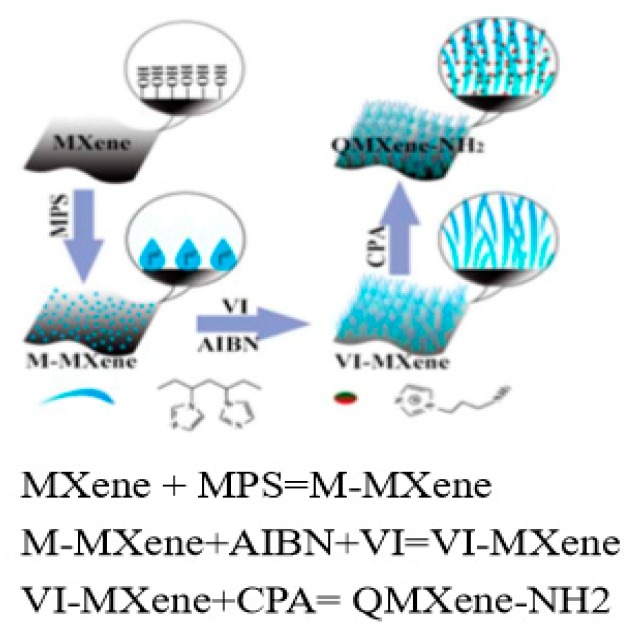
Schematic diagram for preparation process of QMXene-NH_2_.

(1) Preparation of MXene: First, TiH_2_, TiC, and Al were completely mixed in a molar ratio of 1:2:1.2. Then, the powders were added in an alundum crucible, compacted, and put into a tube furnace that was heated to 1450 °C at the speed of 10 °C/min and kept constant for 2 h, yielding a solid MAX phase. Finally, the MAX powders (325-mesh) were added into 49% HF solution and completely etched by continuous stirring at 60 °C for 72 h. Subsequently, the powders were washed to neutral by using deionized water and thoroughly dried into MXene lamellar material.

(2) Preparation of functionalized MXene (Figure 1): Dried MXene, absolute ethanol, and ammonia water were added into a 250 mL round-bottomed flask, homogeneously distributed by ultrasonication for 4 h, and continuously stirred for 24 h. The mixture was ultrasonicated at 200 W for 30 min, combined with 1 mL of Modified polystyrene (MPS), continuously stirred for 32 h, centrifuged with absolute ethanol, washed, and dried into M-MXene. Afterwards, M-MXene (0.6 g) and acetonitrile (80 mL) were added into a 100 mL one-necked flask and dispersed by 6 h of ultrasonication. After AIBN (0.018 g) and 1-vinylimidazole (0.6 mL) were completely dispersed by ultrasonication in an ice bath for 20 min, the mixture was distilled at 82 °C for 30 min, centrifuged, washed, and dried into MXene-containing imidazole brushes on the surface (VI-MXene). Then VI-MXene (0.6 g) and chloropropylamine (0.015 mmol) were added into absolute ethanol (150 mL), dispersed by continuous ultrasonication for 4 h, and refluxed at 80 °C for 24 h. The resulting product was centrifuged with absolute ethanol, washed, and dried into imidazolium brush-functionalized MXene (QMXene-NH_2_).

(3) Preparation of hybrid membrane: Appropriate amounts of QMXene-NH_2_ and CS were dissolved in glacial acetic acid solution (2.5%, 60 mL), combined with 25% glutaraldehyde solution (0.2 mL), and further stirred for 3 h. The membrane casting solution was thereafter slowly cast onto a levelled glass plate and dried at room temperature until the solvent completely volatilized. Finally, the resulting membrane was subjected to ion exchange by soaking in 1 M KOH solution for 24 h and residual KOH was removed by soaking in deionized water. The prepared membrane was referred to as CS/QMXene-NH_2_-*X*, where *X* is the mass percentage of filler material to CS (*X* = 2.5, 5, 7.5, and 10).

(4) The OH^−^ conductivity across the membrane was measured in a conductivity cell by the alternating current (AC) impedance spectroscopy method. The membrane resistance was tested by two-point probe alternating current impedance spectroscopy with an oscillating voltage of 20 mV over a frequency range of 106–10 Hz using an electrode system connected with a frequency response analyzer. Recently, a practical and reproducible ex situ method for measuring the true value of the hydroxide conductivity of AEMs was reported. This method offered excellent reproducibility compared with the methods currently used to measure OH^−^ conductivity. We will try this method of measuring conductivity in the following work [22].

## 3. Results and Discussion

### 3.1. Experimental Results

The addition of functionalized MXene to the chitosan membrane significantly enhanced the thermal stability and mechanical properties of the chitosan membrane. The hybrid membrane became more thermally stable due to a delay of degradation at the second stage (230–320 °C), and the tensile strength of the hybrid membrane was significantly raised to 29.2–41.0 MPa. In addition, the functionalized MXene with high specific surface area constructed a continuous ion transport channel inside the membrane, which made the OH^−^ conductivity of the hybrid membrane significantly improved.

### 3.2. Characterizations of Functionalized MXene

**Figure 2:**
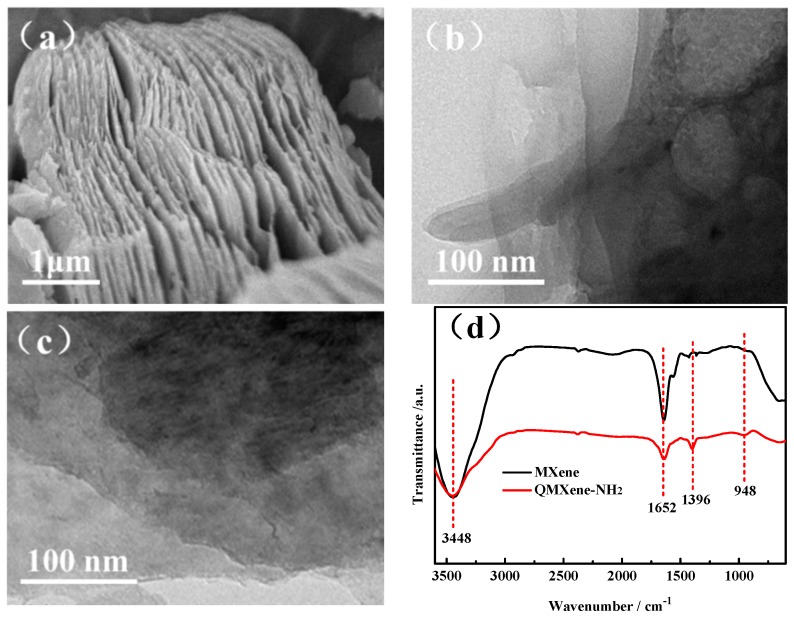
SEM images of MXene (**a**); TEM images (**b**,**c**) and FTIR spectra (**d**) of MXene and QMXene-NH_2_.

For organic–inorganic composite membranes, the material matching was very important, which determined the compatibility between the two phases and the continuity of ion conduction. In this study, the surface of MXene sheet was modified to enhance the interfacial compatibility between inorganic and organic polymers and the defect free composite membrane was prepared. This was a commonly used treatment method for hybrid membranes. The SEM image of MXene was shown in Figure 2a. The results of SEM showed that the two phases were compatible. Clearly, MXene had a two-dimensional lamellar structure with high rigidity. After simple dispersion by ultrasonication, mono-lamellar MXene was obtained and its TEM image is exhibited in Figure 2b. MXene maintained an intact structure after dispersion and had a light-colored surface. After modification, the color of QMXene-NH_2_ (Figure 2c) deepened owing to imidazolium brushes. Since MXene was etched from Ti_3_AlC_2_, its surface contained abundant –OH as well as small amounts of –F and –O–. Therefore, we characterized the chemical structures of MXene before and after surface modification by FTIR spectroscopy (Figure 2d). MXene exhibits vibration peaks of –OH at 3448 cm^−1^ and 1652 cm^−1^ and a stretching vibration peak of Ti-O-Ti at 500–750 cm^−1^ [23]. After functionalization, –OH and –O– on the surface of MXene participated in reaction, so the intensities of these peaks plummet. The characteristic peaks of imidazolium were overlapped by the strong ones of MXene. Regardless, there was a stretching vibration peak of N-H (–NH_2_) in QMXene-NH_2_ at 3000–3500 cm^−1^ [24], suggesting that the imidazolium group had been successfully introduced.

**Figure 3:**
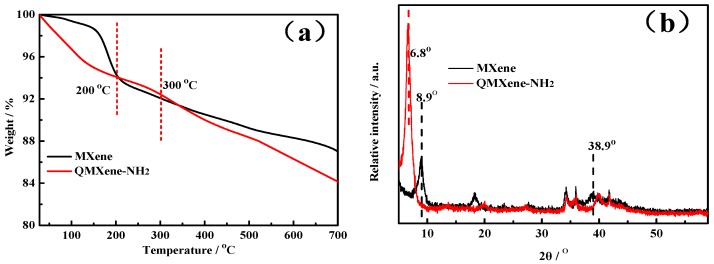
TGA (**a**) and XRD (**b**) curves of MXene and QMXene-NH_2_.

The TGA curves of MXene and QMXene-NH_2_ are shown in Figure 3a. As an inorganic ceramic material, MXene has high thermal stability and its thermal degradation can be divided into two main stages: (1) Weight loss of only 6% from room temperature to 200 °C, mainly originating from water molecules adsorbed on the surface and between lamellas of MXene; and (2) weight loss at above 200 °C, mainly owing to the degradation of –OH on the MXene surface [25]. Notably, the weight loss of QMXene-NH_2_ from 25 to 200 °C exceeded that of MXene, primarily because more free water molecules were adsorbed by hydrophilic imidazolium brushes on the surface of the former, which was accelerated with rising temperature. After functionalization, –OH on the MXene surface was replaced by Si-O-Ti, thus enhancing the thermal stability and reducing the weight loss. Strasser et al. reported that imidazolium cations started to degrade at approximately 300 °C [26]. Accordingly, the thermal degradation of QMXene-NH_2_ was apparently accelerated at over 300 °C. In addition, combined with TGA data, after weight loss the remaining mass was less than 45%, which was within its theoretical range as stated in the reported literature [27]. Therefore, there was no carbonization in the temperature range we tested.

The structure of MXene after surface modification was further characterized by XRD (Figure 3b). Since MXene was etched from Ti_3_AlC_2_, there are two characteristic peaks at 8.9° (002 crystal plane) and 38.9° (104 crystal plane) [28]. The interlamellar spacing of MXene was calculated as 0.99 nm by the Bragg equation (d = *λ*/2sin*θ*, *λ* = 1.54 Å). Surface modification of 2*θ* shifts to 6.8° corresponded to an interlamellar spacing of 1.29 nm. Such increase can mainly be ascribed to weakened hydrogen bonding between lamellas of MXene by ion brushes. In addition, the introduction of an imidazolium cation brush on the surface of the MXene still maintained an intrinsic lamellar structure that might facilitate the construction of a continuous ion transport channel within the membrane as reported in the literature.

### 3.3. Microstructure, Physical, and Chemical Properties of Membrane

**Figure 4:**
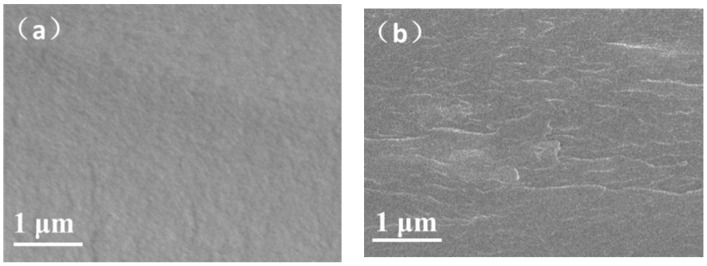
SEM images of cross-sections of (**a**) CS and (**b**) CS/QMXene-NH_2_-7.5.

A hybrid alkaline anion exchange membrane was fabricated by mixing imidazolium brush-functionalized MXene and CS matrix and its cross-section is presented in Figure 4. The control CS membrane (Figure 4a) had a flat and smooth cross-section without any defect. Filling CS matrix with QMXene-NH_2_ generated mild wrinkles, primarily because nano lamellas interfered with the configuration of CS chain segments. Unlike rod-like or spherical materials, functionalized MXene did not form curls or large particles inside the membrane, so bulge was barely visible. QMXene-NH_2_ was compatible with CS matrix, without obvious interfacial defect. Because QMXene-NH_2_ was uniformly dispersed in CS matrix, it was considered that it might benefit the construction of continuous OH^−^ conduction channels.

**Figure 5:**
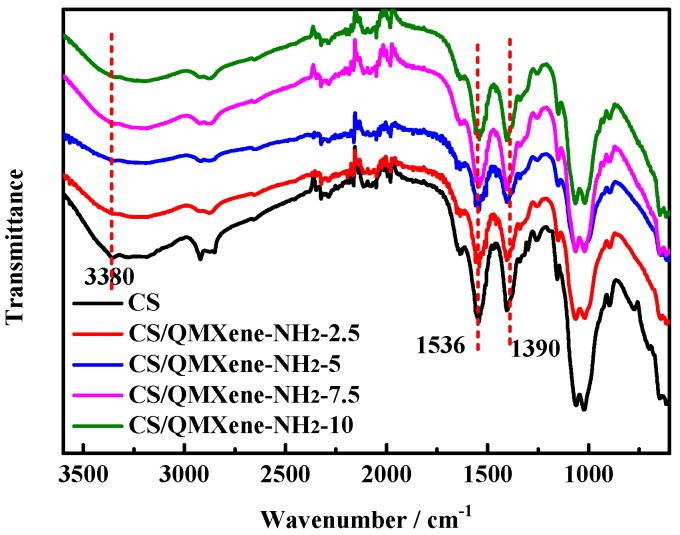
FTIR spectra of control CS and CS/QMXene-NH_2_ membranes.

The FTIR spectra of control CS (Chitosan membrane) and hybrid membranes are displayed in Figure 5. The control CS membrane exhibits characteristic peaks of amide band I, band II, and band III at 1633 cm^−1^, 1536 cm^−1^, and 1390 cm^−1^ respectively [29,30,31]. The peak intensities of CS/QMXene-NH_2_ all decrease, mainly because two-dimensional lamellar material interfered with the IR absorptions of groups on CS chain segments. In addition, the interference was further enhanced with increasing filler amount, which gradually reduced the absorptions. The broad peaks of all membranes at 3380 cm^−1^ correspond to water adsorbed inside the CS membrane [32]. Introducing two-dimensional lamellar material interfered with the configuration and decreased the motility of CS chain segments, leading to peak intensity reduction of the hybrid membrane.

**Figure 6:**
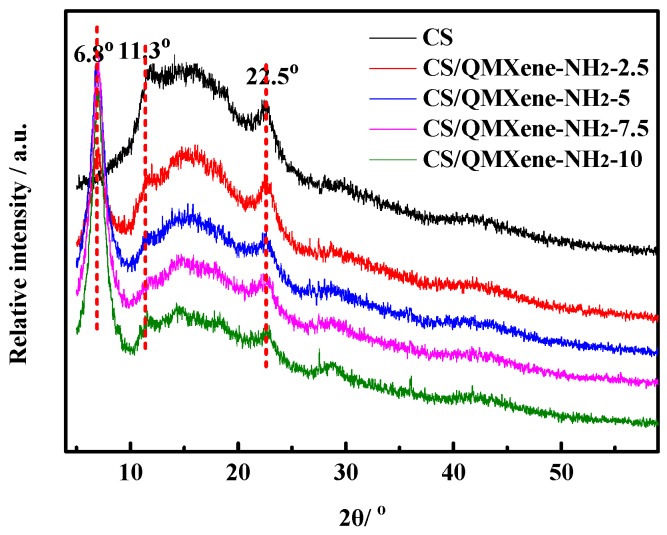
XRD patterns of control CS and CS/QMXene-NH_2_ membranes.

For an ion exchange membrane, the degree of crystallinity of polymer chain segments significantly affected ion conduction. Generally, polymer crystallization hindered ion conduction which, when weakened, promoted the conduction process. The XRD patterns of control CS and hybrid membranes are shown in Figure 6. The crystalline peaks of the control CS membrane are located at 2*θ* of 11.3° and 22.5° [33]. In contrast, the hybrid membrane has a characteristic peak of QMXene-NH_2_ at 6.8°, accompanied by a decreased degree of crystallinity owing to the spatial interference of QMXene-NH_2_. It was known that the configuration of CS chain segments affected the crystallinity of polymer chain segments. However, the addition of two-dimensional lamellar materials just interfered with the configuration of CS chain segments, which reduced the crystallinity of the hybrid membrane. So, as the amount of QMXene-NH_2_ was elevated, the configuration of CS chain segments was further disrupted, lowering the degree of crystallinity of the hybrid membrane as a result.

**Figure 7:**
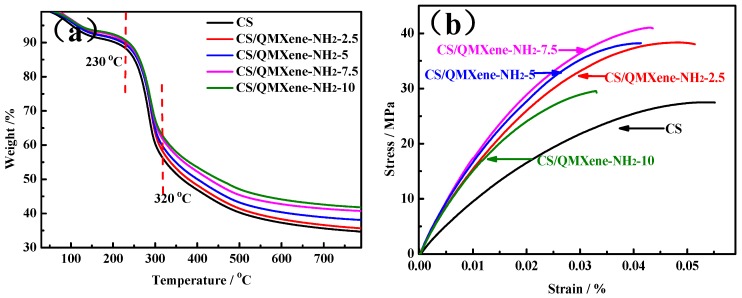
(**a**) TGA curves and (**b**) stress-strain curves of control CS and CS/QMXene-NH_2_ membranes.

The addition of inorganic compounds to the CS introduced the inorganic particles, which improved the organic network structure, enhanced mechanical properties, and improved thermal stability. The thermal stability of control CS and hybrid membranes was tested (Figure 7a). Containing considerable –NH_2_ and –OH, CS is highly hydrophilic, so the moisture loss reached 8.3% at the first stage (25–130 °C). The CS side chain degraded mainly at the second stage (230–320 °C), and the backbone did so at the third stage (320–800 °C) [34]. Compared with control CS membrane, adding QMXene-NH_2_ reinforced the interfering effects of CS chain segments, which then reduced the hydrophilicity of the hybrid membrane. Hence, the moisture loss at the first stage (25–130 °C) was lower. With a large lamellar structure, QMXene-NH_2_ strongly interfered with the motility of CS chain segments. As a result, the hybrid membrane became more thermally stable due to the delay of degradation at the second stage (230–320 °C). With rising filling amounts of QMXene-NH_2_, the ash content of the hybrid membrane gradually increased at the third stage (320–800 °C).

Additionally, the tensile properties of control CS and hybrid membranes were detected (Figure 7b). CS was a semi-crystalline polymer. Herein, the control CS membrane had a tensile strength of 27.5 MPa, a breaking elongation of 5.5%, and a Young’s modulus of 920.2 MPa. After the addition of QMXene-NH_2_ with high mechanical strength as an inorganic lamellar material, the tensile strength of the hybrid membrane was significantly raised to 29.2–41.0 MPa. Nevertheless, since the inorganic lamellar material had poor toughness, the breaking elongation gradually dropped with increasing filling amounts.

### 3.4. Ion Exchange Capacity (IEC), Water Uptake Ratio, and Swelling Ratio

**Figure 8:**
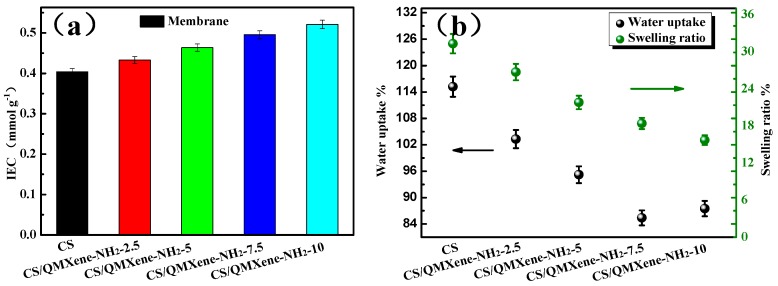
Filler content-dependent IEC values (**a**), water uptake ratios and swelling ratios (**b**) of control CS and CS/ CS/QMXene-NH_2_ membranes.

The IEC of ion exchange membranes can reflect the number of exchangeable OH^−^ inside, determining the OH^−^ conductivity of alkaline membranes to a certain extent. The IEC values of control CS and hybrid membranes are shown in Figure 8a. According to functionalized QMXene-NH_2_ content in CS (CS/QMXene-NH_2_-2.5, CS/QMXene-NH_2_-5, CS/QMXene-NH_2_-7.5, and CS/QMXene-NH_2_-10), the IEC of the four membranes were 0.43 mmol g^−1^, 0.46 mmol g^−1^, 0.49 mmol g^−1^, and 0.51 mmol g^−1^ respectively. Rich in –NH_2_, CS undergoes protonation in the presence of water, thereby producing OH^−^. The control CS membrane had an IEC of 0.40 mmol g^−1^, close to that reported by Wan et al. [35]. Given that QMXene-NH_2_ had abundant imidazolium groups on the surface, filling it into CS matrix markedly increased IEC of the hybrid membrane. For instance, IEC reached as high as 0.49 mmol g^−1^ at the QMXene-NH_2_ filling amount of 7.5%.

Meanwhile, the water uptake ratios of control CS and hybrid membranes at 20 °C were measured (Figure 8b). Containing large amounts of hydrophilic –OH and –NH_2_, CS chain segments provided a high water uptake ratio of 115.2% for the control membrane. Moreover, water molecules exerted strong solvation effects on highly hydrophilic CS chain segments, resulting in apparent swelling of the membrane. At 20 °C, the swelling ratio of the control CS membrane was 31.3%. QMXene-NH_2_ blocked the motility of CS chain segments through evident spatial interference, so the water uptake ratio and swelling ratio of the hybrid membrane both dropped. For example, when 7.5% QMXene-NH_2_ was filled, the two values reduced to 85.4% and 18.4% respectively. The water permeation was much higher than that of the most similar membranes as reported [36].

### 3.5. Hydroxide Conductivity of Membrane

**Figure 9:**
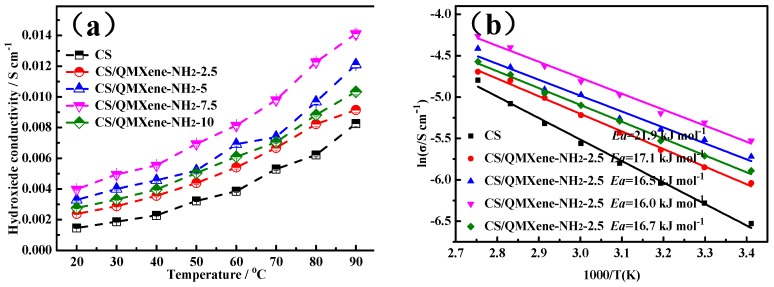
(**a**) Temperature-dependent OH^−^ conductivity and (**b**) Arrhenius plots of OH^−^ conductivity of control CS and CS/QMXene-NH_2_ membranes.

OH^−^ conductivity was the most important index for evaluating the performance of alkaline membranes, determining the practical applicability in preparing alkaline fuel cells. The conductivity of the control CS membrane at 20 °C and 100% RH reached 0.00146 S cm^−1^ as shown in Figure 9a, being comparable to that reported before [37]. Addition of QMXene-NH_2_ constructed continuous conduction channels (imidazolium brushes and interfacial water channels) inside the CS membrane, which increased significantly the OH^−^ conductivity of the hybrid membrane. For instance, when the filling amount was 2.5%, 5%, and 7.5%, the conductivity of CS/QMXene-NH_2_ at 100% RH was raised with addition of QMXene-NH_2_ as shown in Figure 9a. The conductivity of CS/QMXene-NH_2_ with addition of 7.5% QMXene-NH_2_ at 100% RH was raised 172% to 0.00398 S cm^−1^ compared with CS (0.00146 S cm^−1^ Figure 9a). The addition of 7.5%QMXene-NH_2_ was enough for alkaline fuel cell application, because further elevating the content of QMXene-NH_2_ (10%) induced lamellar agglomeration and undermined the continuity of interfacial channels, eventually decreasing the OH^−^ conductivity. With rising temperature, the OH^−^ conductivity gradually increased, mainly because: (1) Such conduction was a thermal control process so the motility of OH^−^ was promoted as the temperature rose; (2) water molecules were more capable of transport, diffusion, and migration due to facilitated motility by temperature elevation; and (3) enhancement of the motility of CS chain segments decreased the energy barrier of OH^−^ conduction [38]. The Arrhenius plots of control CS and hybrid membranes are presented in Figure 9b. The activation energy of OH^−^ conduction of the control CS membrane at 100% RH was 21.9 kJ mol^−1^ and the conduction energy barrier plummeted after addition of QMXene. For example, the OH^−^ conductivity of the hybrid membrane dropped to 16.0 kJ mol^−1^ at the filling amount of 7.5%.

## 4. Conclusions

(1) QMXene-NH_2_ and CS had high interfacial compatibility and the former could be homogeneously dispersed in CS matrix.
(2) By constructing continuous OH^−^ conduction channels inside the membrane, QMXene-NH_2_ increased the number of carrier sites allowing ion jumping and conduction.
(3) Adding QMXene-NH_2_ significantly augmented the OH^−^ conductivity of the hybrid membrane. At 20 °C, such conductivity of the hybrid membrane (3.98 mS cm^−1^) was 2.72-fold that of the control CS membrane.
(4) Adding QMXene-NH_2_ boosted both the thermal and mechanical stabilities and reduced the membrane swelling ratio.
(5) The addition of QMXene-NH_2_ significantly increased the ion exchange capacity of the hybrid membrane and the water uptake ratio and swelling ratio of the hybrid membrane both dropped. For example, when 7.5% QMXene-NH_2_ was filled, the two values reduced to 85.4% and 18.4% respectively.

In summary, we herein prepared a novel functionalized nanolamellar material that effectively enhanced the overall performance of CS membranes. Hence, it is a feasibly applicable anion exchange membrane.
